# Promising therapeutic effect of thapsigargin nanoparticles on chronic kidney disease through the activation of Nrf2 and FoxO1

**DOI:** 10.18632/aging.102437

**Published:** 2019-11-12

**Authors:** Fong-Yu Cheng, Yu-Hsuan Lee, Yung-Ho Hsu, I-Jen Chiu, Yu-Jhe Chiu, Yuh-Feng Lin, Hui-Wen Chiu

**Affiliations:** 1 Department of Chemistry, Chinese Culture University, Taipei, Taiwan; 2 Department of Cosmeceutics, China Medical University, Taichung, Taiwan; 3 Department of Food Safety/Hygiene and Risk Management, College of Medicine, National Cheng Kung University, Tainan, Taiwan; 4 Division of Nephrology, Department of Internal Medicine, Shuang Ho Hospital, Taipei Medical University, New Taipei City, Taiwan; 5 Division of Nephrology, Department of Internal Medicine, School of Medicine, College of Medicine, Taipei Medical University, Taipei, Taiwan; 6 Graduate Institute of Clinical Medicine, College of Medicine, Taipei Medical University, Taipei, Taiwan

**Keywords:** endoplasmic reticulum stress, autophagy, FoxO1, Nrf2, chronic kidney disease

## Abstract

Pathophysiological states cause misfolded protein accumulation in the endoplasmic reticulum (ER). Then, ER stress and the unfolded protein response (UPR) are activated. Targeting ER stress may enhance the adaptive UPR and then protect the cell against pathogenic environments. In the present study, we utilized nanotechnology to synthesize thapsigargin nanoparticles (TG NPs) which induced ER stress and the UPR pathway, to study the role of ER stress and autophagy in chronic kidney disease (CKD). We found that the mRNA levels of ER stress- and autophagy-related molecules were elevated in the renal tissue of CKD patients compared to those of healthy individuals. Furthermore, TG NPs induced the UPR pathway and autophagy in HK-2 human kidney tubular epithelial cells. TG NPs protected HK-2 cells against oxidative stress-induced cell death through the activation of Nrf2 and FoxO1. The siRNA-mediated inhibition of Nrf2 or FoxO1 resulted in enhanced oxidative stress-induced cytotoxicity in HK-2 cells. In a mouse model of adenine diet-induced CKD, TG NPs and KIM-1-TG NPs ameliorated renal injury through the stimulation of ER stress and its downstream pathways. Our findings suggest that the induction of ER stress using pharmacological agents may offer a promising therapeutic strategy for preventing or interfering with CKD progression.

## INTRODUCTION

The global prevalence of chronic kidney disease (CKD) is rising in older people and treatment for CKD is important to affect the health of aging population [1]. CKD is a type of kidney disease that causes the gradual loss of kidney function over a period of time and then causes end stage renal disease (ESRD), which requires dialysis or kidney transplantation [2]. CKD leads to irreversible structural degradation in the kidneys, including damage to the tubular epithelium, glomeruli and arterioles [3]. CKD affects millions of people worldwide and is an important public health problem [2]. Therefore, understanding the molecular mechanisms of CKD is a critical challenge for the development of preventive and therapeutic approaches. Previous studies have demonstrated that the endoplasmic reticulum (ER) functions in the regulation of protein homeostasis in the kidneys [4]. The ER has major roles in proteostasis, including the folding, maturation and assembly of proteins [5]. Under pathophysiological conditions, increasing the demand for protein folding or that interfering with normal folding processes leads to misfolded proteins accumulation in the ER and dilatation of the ER. Then, ER stress and the unfolded protein response (UPR) are activated [6, 7]. The UPR has three major signaling pathways including the PRKR-like ER kinase (PERK), inositol-requiring enzyme 1α (IRE1α) and activating transcription factor 6 (ATF6) pathways [4]. Previous studies have indicated that activation of the UPR maintains ER function and may be protective against additional stresses [6, 8]. However, sustained or prolonged ER stress is also cytotoxic and results in apoptosis [7, 9]. Many therapeutic strategies designed to regulate ER stress have been applied in kidney diseases. Low doses of ER stress inducers are protective in some kidney diseases [10, 11]. Recent studies have reported that the PERK pathway of the UPR induces the transcription factor, nuclear factor erythroid-derived-2-related factor-2 (Nrf2) and regulates redox homeostasis to ensure cell survival [12]. Furthermore, PERK can act directly on the transcription factors of the forkhead box, classO (FoxO) to increase FoxO activity [13]. The FoxO family members are key regulators of the cellular stress response and help the cellular antioxidant defense [14]. The kidneys are highly metabolic organs that are rich in oxidation reactions in the mitochondria and susceptible to damage caused by oxidative stress. Several studies have shown that oxidative stress can promote kidney disease progression [15]. Thus, targeting ER stress may enhance the adaptive UPR and thus protect the cell more effectively against pathogenic conditions.

Autophagy is a cellular process for degrading aggregated or long-lived proteins, macromolecules, and organelles [16]. Previous research has shown that autophagy is a precisely regulated process that plays a main role in maintaining cell homeostasis and is a protective response to cellular damage [17, 18]. Ischemic postconditioning, which consists of repeated transient arterial clamping in the early stage of reperfusion, efficiently reduces renal fibrosis and attenuates the degree of epithelial-mesenchymal transition (EMT) after ischemia/reperfusion (I/R) injury by activating autophagy [19]. Another recent study concluded that histone deacetylase inhibitors protect against cisplatin-induced acute kidney injury (AKI) by activating autophagy in proximal tubular cells [20]. ER stress is known to induce autophagy in mammalian cells including renal epithelial cells [18, 21]. Pallet et al. indicated that the powerful immunosuppressive drug cyclosporine (CsA) activates autophagy through ER stress induction as a mechanism to protect against tubular cell death [18]. Evidence is presented indicating that a prior ER stress-induced UPR is capable of conferring cytoprotection in an AKI model [18]. However, the mechanism underlying the promising therapeutic effect of ER stress and the role of ER stress-induced autophagy in CKD are not known.

Nanomaterials (NMs) are defined as materials with internal or surface structures on the nanoscale of 1 to 100 nm. Because of their special physiochemical features, which include large specific surface areas, small size and optical properties, NMs have been used in many scientific areas. Currently, many nanoparticles (NPs) have been utilized in the area of biomedicine for therapeutic and diagnostic purposes [22]. NPs supply potentially efficient and safe selective drug delivery as well as specific biomarker and/or imaging probe binding [23]. Furthermore, NP drug delivery systems not only enhance targeting ability, but also control drug release [24, 25]. Thus, NPs can deliver drugs very effectively to a target site to enhance efficacy and reduce side effects [26]. In recent years, therapeutic NPs for drug delivery in cancer have received much attention. However, there are few published articles utilizing NP drug delivery systems in kidney disease. In the present study, we analyzed changes in ER stress- and autophagy-related genes in the renal tissue of healthy individuals and CKD patients using the Gene Expression Omnibus (GEO) database. Furthermore, we tested whether NPs carrying the ER stress inducer thapsigargin (TG) could enhance autophagy and confer protection against oxidant-induced cell death. Based on previous studies by us and others, kidney injury molecule-1 (KIM-1) is a biomarker for renal proximal tubule injury [27–29]. Therefore, we examined the effect of TG NPs conjugated with an anti-KIM-1 antibody in an adenine-fed mouse model of CKD.

## RESULTS

### The expression profiles of ER stress- and autophagy-related genes in the renal tissue of healthy individuals and CKD patients

**Figure 1:**
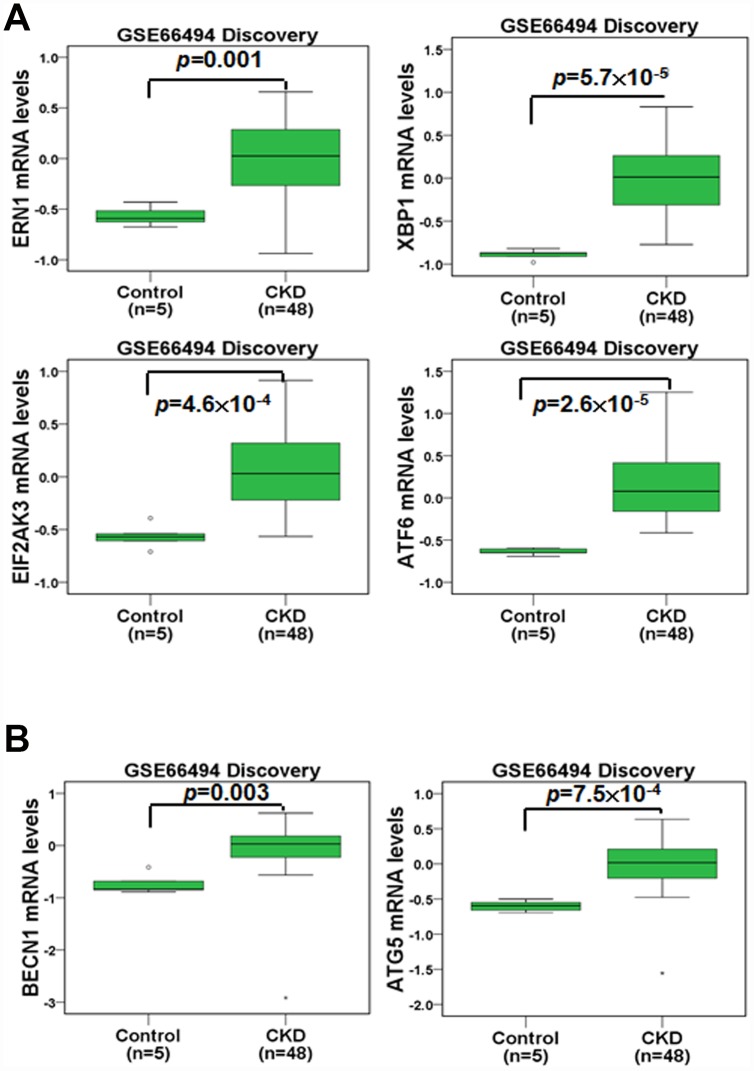
**The mRNA levels of ER stress- and autophagy-related molecules were analyzed in the renal tissue of healthy individuals and CKD patients.** The mRNA levels of *ERN1*, *XBP1*, *EIF2AK3*, *ATF6* (**A**), *BECN1* and *ATG5* (**B**) were evaluated. Statistical differences were analyzed using a two-sample t-test.

We first compared the gene expression profiles in the renal tissue of healthy individuals and CKD patients (Figure 1). In an Agilent microarray analysis, the levels of *ERN1*, *XBP1*, *EIF2AK3* and *ATF6* were predominantly upregulated in the renal tissue of the CKD patients compared to that of the healthy individuals in the discovery set (Figure 1A). These genes are involved in ER stress pathways (UPR pathways). Furthermore, we analyzed the expression profiles of autophagy-related genes in the renal tissue of the healthy individuals and CKD patients. The results indicated that the expression of *BECN1* and *ATG5* was elevated in the renal tissue of the CKD patients in the discovery set (Figure 1B). In addition, the gene changes in the validation set agreed with those of the discovery set (Supplementary Figure 1). These results showed that the regulation of ER stress and autophagy may participate in CKD progression.

### Physical characterization, entrapment efficiency and drug loading of TG NPs and KIM-1-TG NPs

**Figure 2:**
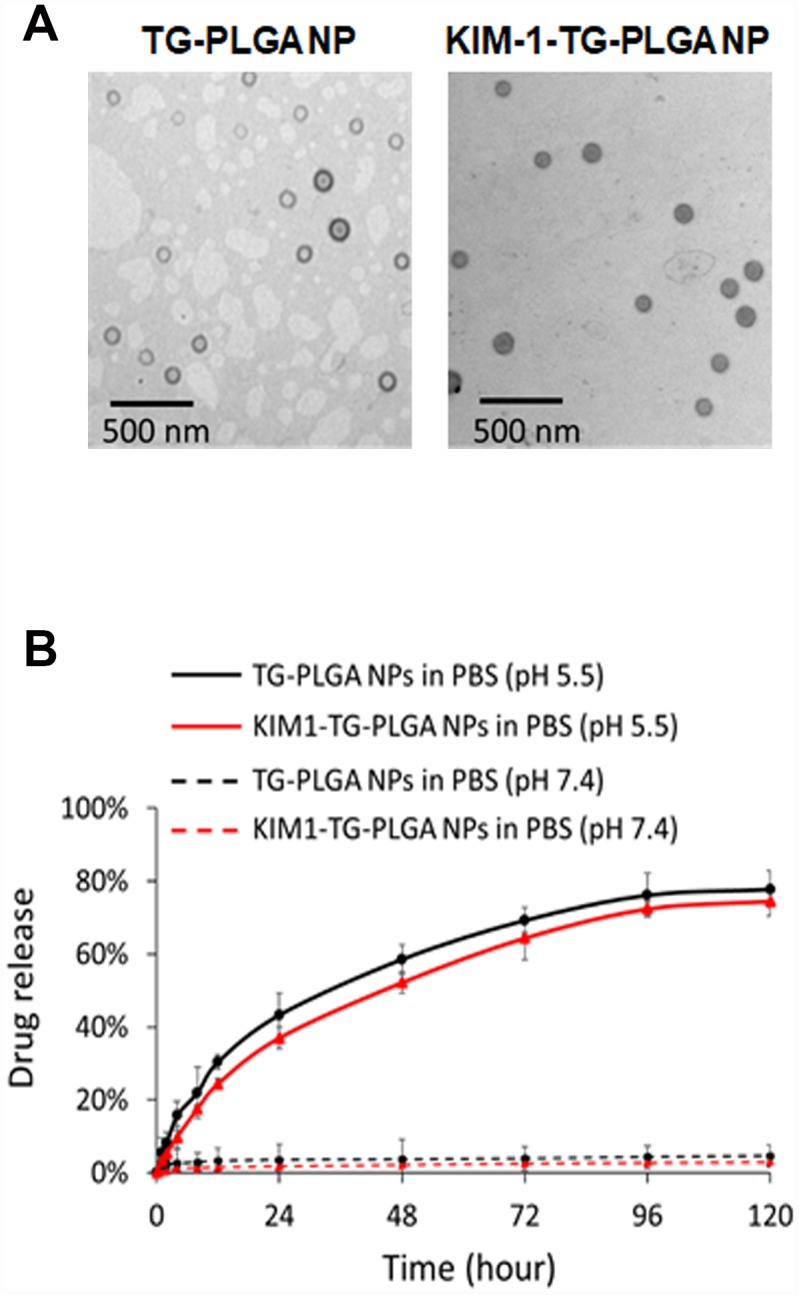
**The morphology and TG release profile of TG NPs and KIM-1-TG NPs.** (**A**) TEM images of the TG NPs and KIM-1-TG NPs. (**B**) *In vitro* TG release profile of the TG NPs and KIM-1-TG NPs incubated at 37°C in PBS (pH 5.5 and 7.4).

TEM images of the TG NPs and KIM-1-TG NPs are shown in Figure 2A. The diameter of the TG NPs was ~90.1 nm. The hydrodynamic diameter and zeta-potential of the TG NPs were 110.5 nm and -36.3 mV in water, respectively (Table 1). The efficiency of TG entrapment was approximately 80%. The amount of TG in the PLGA NPs (w/w) was approximately 34 μg per mg of PLGA. The loading efficiency of TG in the PLGA NPs was ~34% (Table 1). The TG release from the TG-PLGA NPs dissolved in PBS, pH 7.4 and 5.5 (10 mM, pH 5.5 and 7.4) at 37°C is shown in Figure 2B. The saturated release of TG was close to 78% in PBS at pH 5.5 within 96 h but only ~5% in PBS at pH 7.4 within 120 h. These results indicate that TG was efficiently trapped in PBS at pH 7.4 but could be released in PBS at pH 5.5 which mimic the uptake of NPs by cells.

**Table 1:** Basic characteristics of poly(lactic-co-glycolic acid) NP, TG-loaded NP and KIM-1-TG-loaded NP.

	**PLGA NP**	**TG-PLGA NP**	**KIM-1-TG-PLGA NP**
**Hydrodynamic diameter (nm)**	105.2	110.5	200.4
**PDI**	0.204	0.234	0.221
**Zeta potential (mV)**	−35.2	−36.3	−38.6
**Real diameter (nm)**		90.1	91.3
**Encapsulation efficiency (μg TG/ mg PLGA)**		34	34

PLGA: Poly(lactic-coglycolic acid); TG NP: Thapsigargin-encapsulated PLGA nanoparticle; KIM-1: Kidney injury molecule-1; KIM-1-TG NP: KIM-1 antibody-conjugated TG-PLGA NP; PDI: Polydispersity index.

For the purpose of targeted therapy, antibody-conjugated TG NPs (KIM-1-TG NPs) were synthesized, and their hydrodynamic diameter and zeta-potential were 200.4 nm and −38.6 mV, respectively (Table 1). The behaviors of the TG release from the KIM-1-TG NPs were similar to those from the TG NPs dissolved in PBS, pH 7.4 and 5.5 at 37°C (Figure 2B). The saturated release of TG from the KIM-1-TG NPs was close to 74.5% in PBS at pH 5.5 within 96 h but only ~3% in PBS at pH 7.4 within 120 h. These results also indicate that TG was efficiently trapped in PBS at pH 7.4 and could be released in PBS at pH 5.5.

### Viability and ER stress of the human kidney tubular epithelial cell line HK-2 treated with TG NPs

**Figure 3:**
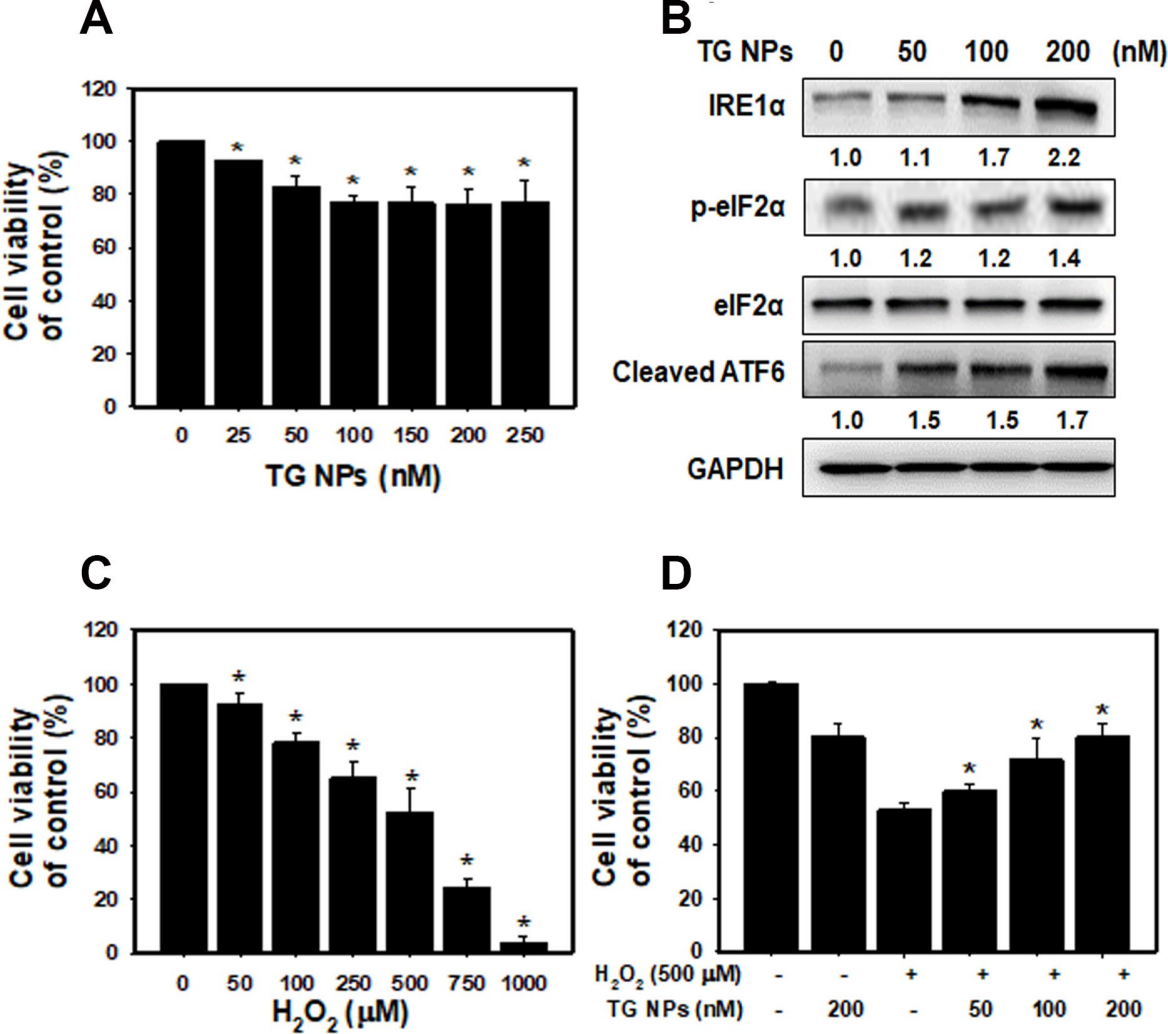
**The cell viability and UPR-related protein expression after TG NP treatment in HK-2 cells.** (**A**) Cell viability was analyzed by SRB assays. Cells were treated with different concentrations of the TG NPs for 24 h. *p < 0.05 versus the control. (**B**) UPR-related protein expression was measured by western blot analysis. Cells were treated with different concentrations of the TG NPs for 24 h. (**C**) Cell viability after H_2_O_2_ treatment was analyzed by SRB assays. Cells were treated with different concentrations of H_2_O_2_ for 18 h. *p < 0.05 versus the control. (**D**) TG NPs showed cytoprotection against oxidative stress. HK-2 cells were pretreated with or without the TG NPs for 6 h and then incubated with H_2_O_2_ for 18 h. *p < 0.05 versus the H_2_O_2_ group.

After treatment with TG NPs for 24 h, cell viability was decreased in HK-2 cells as examined by SRB assays (Figure 3A). However, high concentrations of the TG NPs (200 and 250 nM) only reduced the cell viability to approximately 80%. These results provide evidence that TG NPs exhibited little cytotoxic effects on human kidney proximal tubular epithelial cells. A previous report showed that TG is an ER stress inducer in renal epithelial cells [30]. Next, we investigated whether TG NPs induce ER stress in HK-2 cells. The expression levels of UPR-related proteins, including IRE1α, phosphorylated eIF2α and cleaved ATF6, increased with TG NP treatment (Figure 3B). It has been reported that the activation of the UPR during oxidative stress is an adaptive response to maintain cell function and survival. The UPR pathway also increases the expression of antioxidative stress-related genes [31]. We next evaluated the effect of prior ER stress on hydrogen peroxide (H_2_O_2_)-induced cell injury. H_2_O_2_ caused kidney cell cytotoxicity in a concentration-dependent manner (Figure 3C). The selected concentration of H_2_O_2_ caused cell death in approximately 50% of the cells at a dose of 500 μM. As shown in Figure 3D, TG NPs protected HK-2 cells against H_2_O_2_-induced cell death.

### TG NPs induce autophagy in human kidney tubular epithelial cells

**Figure 4:**
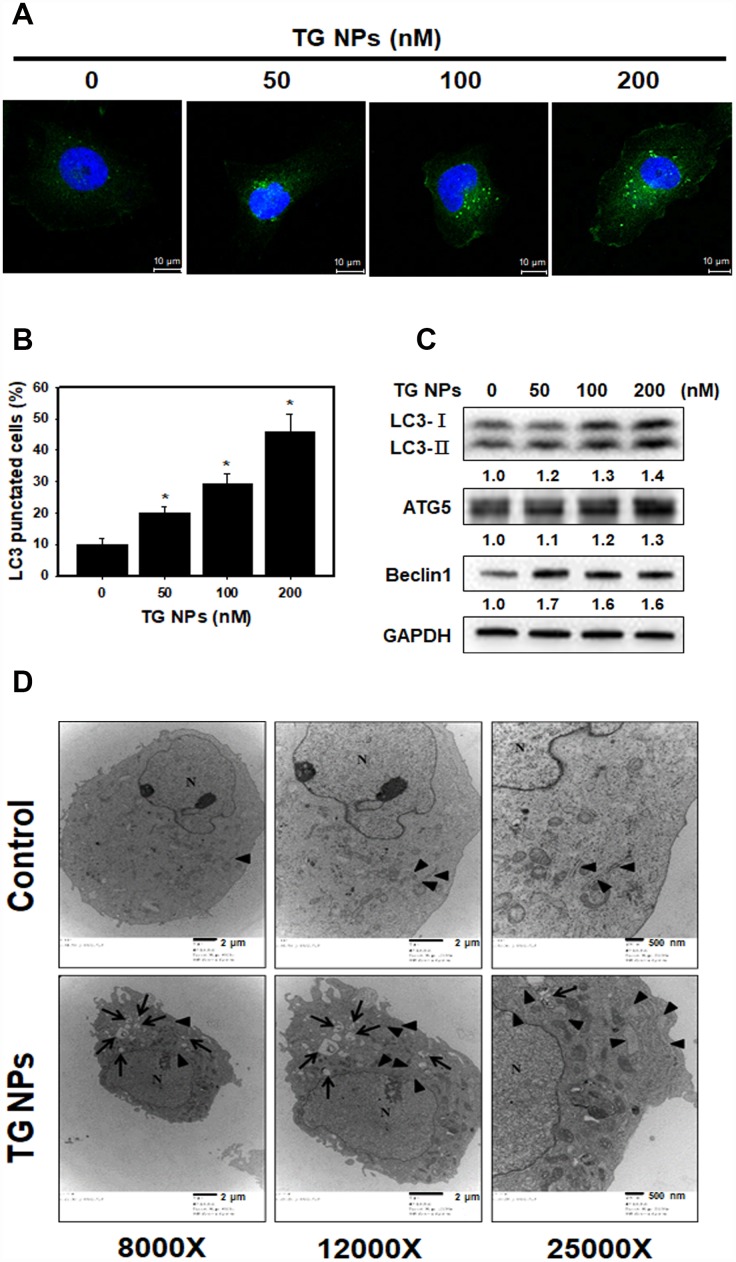
**TG NPs induce autophagy in HK-2 cells.** (**A**) The immunofluorescence staining for the LC3 protein in HK-2 cells treated with the TG NPs was observed using confocal microscopy. LC3 was detected with DyLight™ 488-conjugated secondary antibodies (green), and nuclei were stained with DAPI (blue). (**B**) Quantitative data calculating the number of LC3 dots are shown. Cells were treated with TG NPs for 24 h. *p < 0.05 versus the control. (**C**) Autophagy-related protein expression was measured by western blotting. Cells were treated with different concentrations of the TG NPs for 24 h. (**D**) Cell ultrastructure was observed using TEM. Cells were treated with TG NPs (100 nM) for 24 h. The arrows indicate autophagosomes and autolysosomes. The arrowheads indicate the ER. N indicates the nucleus.

To further determine the occurrence of autophagy, the effect of TG NPs on LC3 (a marker of autophagy) puncta was investigated with immunofluorescence staining. Our data showed that the percentage of LC3 punctate cells was significantly increased in the TG NP treatment group (Figure 4A and 4B). Furthermore, autophagy-related proteins were evaluated by western blot analysis. LC3 is a specific marker of autophagy and exists in two forms, cytosolic LC3-I, which is derived from LC3-I by proteolysis and lipid modification, and membrane-bound LC3-II. The LC3-II level is directly correlated with the number of autophagosomes [32]. The results revealed that the TG NPs reinforced the LC3-II, Atg5 and beclin 1 protein levels (Figure 4C). We also assessed the morphology of HK-2 cells at the electron microscopic level because electron microscopy is the gold standard for monitoring autophagy [33]. As shown in Figure 4D, the cells treated with TG NPs showed an increased number of autophagic vacuoles in the cytoplasmic area compared to the control cells. In addition, TG NPs caused extensive dilatation of the ER. These results indicate that the TG NPs induced ER stress and autophagy in human kidney tubular epithelial cells.

### TG NPs activate Nrf2 and FoxO1 to protect against H_2_O_2_-induced cell death

**Figure 5:**
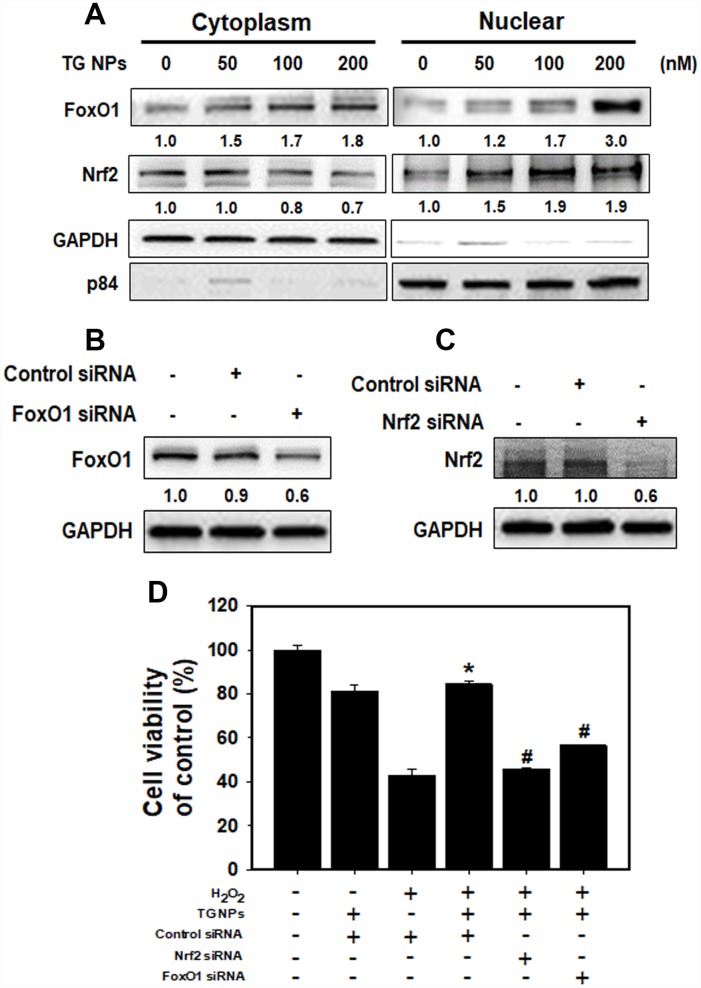
**The roles of FoxO1 and Nrf2 after TG NP treatment in HK-2 cells.** (**A**) The protein levels of FoxO1 and Nrf2 in the cytoplasmic and nuclear fractions of HK-2 cells treated with TG NPs are shown. p84 and GAPDH served as loading controls for the nuclear and cytoplasmic fractions, respectively. Cells were treated with different concentrations of the TG NPs for 24 h. Western blotting showed the efficiency of the siRNA-mediated knockdown of FoxO1 (**B**) and Nrf2 (**C**) expression. (**D**) The effects of FoxO1 or Nrf2 siRNA on cell viability were tested. Cells were transfected with a control, FoxO1-specific or Nrf2-specific siRNA for 24 h, treated with TG NPs (200 nM) for 6 h and then incubated with H_2_O_2_ (500 μM) for 18 h. *p < 0.05, H_2_O_2_+control siRNA versus H_2_O_2_+TG NPs + control siRNA. #p < 0.05, H_2_O_2_+TG NPs + control siRNA versus H_2_O_2_+TG NPs+FoxO1 or Nrf2 siRNA.

To determine whether the TG NPs regulate FoxO1 and Nrf2 translocation, we examined nuclear and cytoplasmic cell fractions (Figure 5A). The results showed that Nrf2 translocation to the cytosol from the nucleus was increased in the HK-2 cells treated with the TG NPs. However, the TG NPs elevated FoxO1 level not only in the cytosol but also in the nucleus. Furthermore, the FoxO1 level of whole cell was increased upon TG NP exposure (Supplementary Figure 2). In addition, we assessed whether TG NPs protect HK-2 cells against H_2_O_2_-induced cell death through the regulation of FoxO1 and Nrf2. Transient siRNA-mediated knockdown of FoxO1 and Nrf2 expression appeared to inhibit FoxO1 and Nrf2 expression, respectively (Figure 5B and 5C). As shown in Figure 5D, the TG NPs reduced H_2_O_2_-induced cell death in HK-2 cells. In contrast, the knockdown of FoxO1 or Nrf2 expression substantially inhibited the protective effect of the TG NPs. Taken together, these results suggest that the TG NPs activated Nrf2 and FoxO1 to protect against H_2_O_2_-induced cell death.

### KIM-1-TG NPs improve adenine diet-induced kidney disease

**Figure 6:**
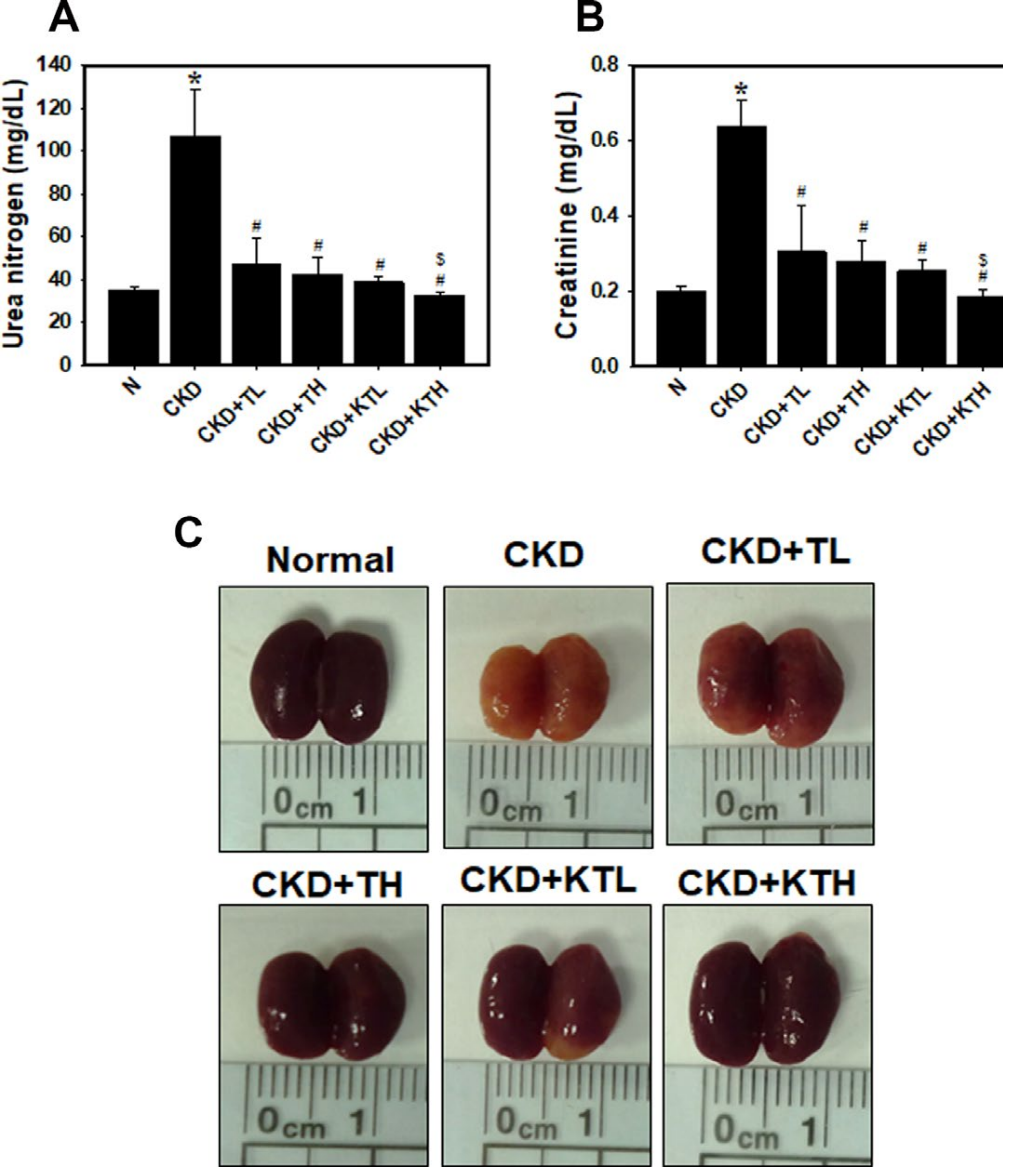
**Serum biochemistry parameters and direct observations of the kidneys in an adenine-induced CKD model following TG NP and KIM-1-TG NP treatment.** (**A**) (**B**) Analysis of serum biochemistry parameters. Creatinine and urea nitrogen levels were assessed after TG NP or KIM-1-TG NP injection. *p < 0.05, normal versus CKD. #p < 0.05 versus CKD. $p < 0.05, CKD+TH versus CKD+KTH. (**C**) Images of an entire kidney. After the experiments, the mice were sacrificed, and the kidneys were removed. N indicates normal group. CKD indicates that mice are adenine-fed and intraperitoneally injected saline. TL indicates low-concentration (0.1 mg/kg) TG NP group. TH indicates high-concentration (0.2 mg/kg) TG NP. KTL indicates low-concentration (0.1 mg/kg) KIM-1-TG NP group. KTH indicates high-concentration (0.2 mg/kg) KIM-1-TG NP group. Details of the mouse model are described in “Materials and Methods”.

**Figure 7:**
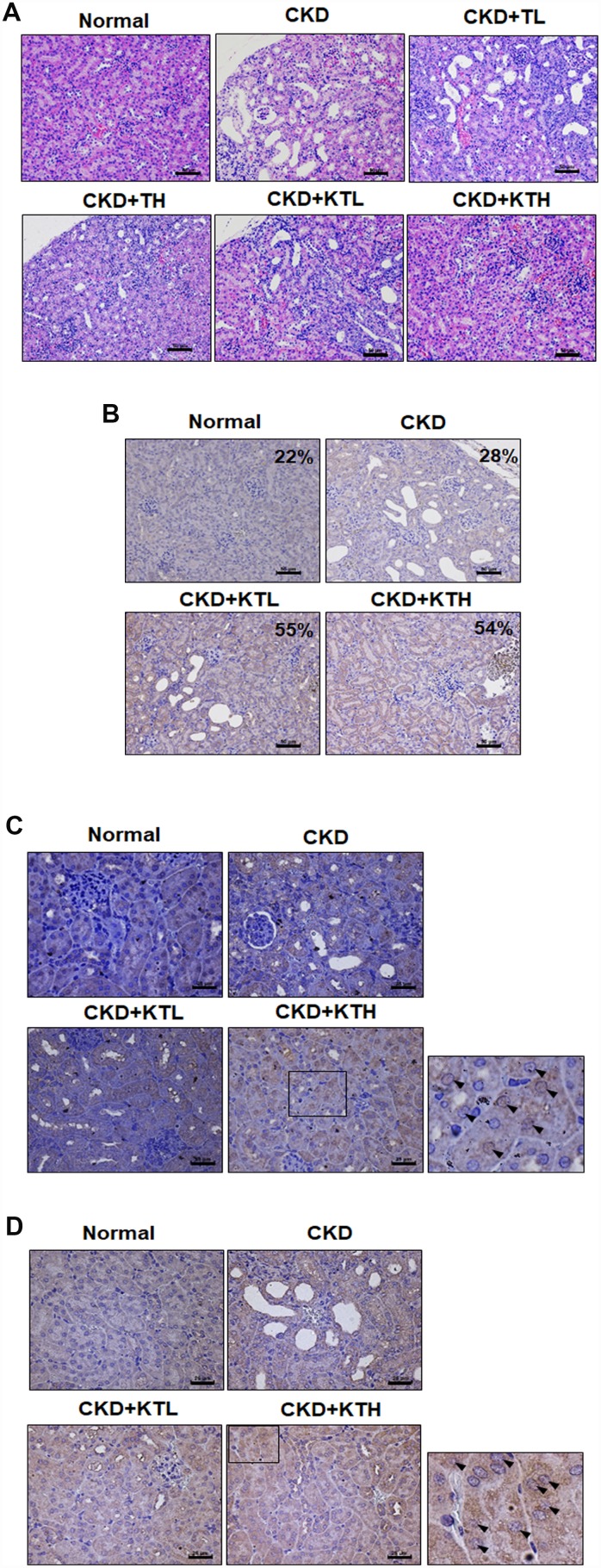
**TG NPs and KIM-1-TG NPs protect against tubulointerstitial injury in mice with CKD induced by adenine.** (**A**) Representative photographs of histological changes showing sections of renal tissue from mice after H&E staining. Immunohistochemical analysis of the protein expression of LC3 (**B**), FoxO1 (**C**) and Nrf2 (**D**) in the sections of renal tissue. The percentage of LC3-positive cells was determined using HistoQuest software (TissueGnostics). (**A**, **B**) Scale bar = 50 μm. (**C**, **D**) Scale bar = 25 μm.

The data accumulated by us and others have revealed that rats or mice continuously fed an adenine-containing diet develop CKD [28, 34, 35]. In the adenine-fed mice (CKD group), there were increases in the serum urea nitrogen and creatinine levels (Figure 6A and 6B). We also found that the groups treated with the TG NPs or KIM-1-TG NPs exhibited significantly reduced nitrogen and creatinine levels compared to the adenine alone group. The urea nitrogen and creatinine levels in mice treated with the high-concentration of KIM-1-TG NPs were lower than those in the mice stimulated with the high-concentration of TG NPs. In addition, the kidneys from the mice were directly observed (Figure 6C). The results showed that the kidney size of the CKD group was significantly smaller than that of the normal group. The kidneys in the CKD group were orange, and the surface of the kidneys was uneven and rough. The kidneys of the TG NP (CKD+TL and CKD+TH) and KIM-1-TG NP groups (CKD+KTL and CKD+KTH) had healthier appearances. The histopathological evaluation of the kidney tissue from the CKD group showed glomerular atrophy, interstitial inflammatory cell infiltration, tubular dilation and loss of the brush border (Figure 7A). However, these abnormalities were greatly ameliorated in the TG NP and KIM-1-TG NP groups. To further explore the effects of the KIM-1-TG NPs on the activation of autophagy, we examined the expression of LC3 in the kidneys from the CKD mice. The results indicated that the KIM-1-TG NP group exhibited increased LC3 expression compared with the CKD group (Figure 7B). In addition, we performed IHC to identify Nrf2 and FoxO1 expression in the kidneys. Immunohistochemical examination revealed that the KIM-1-TG NP group showed increased Nrf2 and FoxO1 immunostaining in the nucleus compared to the CKD group (Figure 7C and 7D). Collectively, these findings suggest that the KIM-1-TG NPs protect against tubulointerstitial injury through the induction of autophagy and activation of the Nrf2 and FoxO1 pathways in the adenine-induced CKD model.

## DISCUSSION

In recent years, nanomaterial-based drug delivery systems have represented potent applications in various disease therapies. NP drug delivery systems enhance the ability to target organs or tissues and ameliorate drug efficacy through slow and sustained release [24, 25]. Furthermore, these functions of NPs can reduce toxicity and side effects [36]. Until now, oncology has been one of major fields of nanomedicine applications. However, there are a few published articles utilizing NP drug delivery systems in kidney disease. In the present study, we synthesized novel NP drugs, KIM-1-TG NPs and TG NPs, which are characterized by high homogeneity and stable suspension in water (Table 1). Moreover, TG was trapped at pH 7.4 and released at pH 5.5 (Figure 2B). The results indicated that TG was only released after the cellular uptake of the TG NPs. We also conjugated an anti-KIM-1 antibody to the TG NPs. KIM-1 is a biomarker for renal proximal tubule injury, and KIM-1 expression is increased in kidney tissue in adenine diet-induced kidney injury [27, 28]. Previously, researchers reported that TG at a dose of 1 mg/kg administered intraperitoneally to mice inhibited renal injury and dysfunction after ischemia-reperfusion [11]. In this study, we used lower doses of the TG NPs and KIM-1-TG NPs (0.1 and 0.2 mg/kg) and greatly decreased adenine-induced kidney damage (Figures 6 and 7A).

The ER offers a special environment that corrects protein folding as nascent polypeptide chains enter the ER lumen. The high concentration of partially folded and unfolded proteins causes aggregation and ER stress [31]. The accumulation of unfolded proteins activates an adaptive signaling pathway (the UPR). Appropriate adaptation to misfolded protein accumulation requires regulation at all levels of gene expression, including transcription, translation and translocation into the ER lumen. All these processes are required to improve proper protein folding and ER homeostasis [31, 37]. The accumulated evidence reveal that modulating ER stress has been utilized in kidney disease. Low doses of ER stress inducers (tunicamycin or TG) are protective in Heymann nephritis [38], ischemic acute kidney injury [11] and mesangioproliferative glomerulonephritis [10]. Wolcott-Rallison disease, which is caused by an inherited defect in the UPR, causes renal insufficiency including proteinuria, podocyte or tubular epithelial cell dysfunction and prerenal azotemia [39, 40]. In the current study, the mRNA levels of molecules in the UPR pathway including ERN1, XBP1, EIF2AK3 and ATF6 were predominantly upregulated in the renal tissue of the CKD patients compared to that of the healthy individuals (Figure 1A and Supplementary Figure 1A). The TG NPs promoted kidney cell survival in H_2_O_2_-induced injury (Figure 3D). In the adenine diet-induced CKD model, the TG NPs and KIM-1-TG NPs greatly ameliorated adenine-induced kidney damage (Figure 6 and 7A). Previous research has shown that PERK of the UPR pathway causes the nuclear translocation of Nrf2 and increases the transcription of Nrf2 target genes. Furthermore, Nrf2 function is a key factor for cell survival following ER stress [41]. Nrf2 controls the induction of phase 2 detoxifying enzymes and genes encoding antioxidant proteins [42]. p62, which serves as a mediator of autophagy, protects against AKI by regulating the Keap1-Nrf2 signalling pathway [43]. In addition, the PERK branch of ER stress can activate FoxO [44]. FoxO proteins are transcription factors that are known to play critical roles in cell differentiation, proliferation and the response to oxidative stress [45]. Previously, researchers reported that autophagy enhancement through increasing of the FoxO1/Rab7 axis may be a potential therapeutic strategy to improve podocyte injury [46]. In our study, the TG NPs induced the nuclear translocation of Nrf2 and increased FoxO1 expression (Figure 5A and Supplementary Figure 2). Moreover, the downregulation of Nrf2 or FoxO1 expression inhibited the cytoprotective effect of the TG NPs (Figure 5D). In the mouse model of CKD, the KIM-1-TG NP group showed increased Nrf2 and FoxO1 immunostaining in the nucleus compared to the CKD group (Figure 7C and 7D).

The accumulated evidence reveals that ER stress can induce autophagy in mammalian cells [18, 47]. A protective role for ER stress-induced autophagy has been reported in cyclosporine-mediated ER stress in renal tubular cells [18]. In another study, ER stress-induced autophagy protected renal tubular epithelial cells from oxidant- and ATP depletion-induced cell death. Autophagy can be attributed to misfolded proteins, and this process can scavenge damaged proteins to maintain ER function [48, 49]. Many studies have indicated that autophagy plays important roles in kidney maintenance, diseases and aging. Autophagy is induced under ischemic injury or toxic forms of injury to the kidneys and appears to be an early stress response [50]. Previous research has shown that the upregulation of autophagy is protective against kidney disease. Cisplatin-induced autophagy in renal tubular epithelial cells acts as a prosurvival mechanism against cell apoptosis [51]. In addition to its pro-survival role, autophagy may also contribute to cell death in some cells depending on the stimulus [52, 53]. Our studies showed that the expression of autophagy-related genes (*BECN1* and *ATG5*) was elevated in the renal tissue of the CKD patients (Figure 1B). Furthermore, TG NPs increased the number of LC3 puncta and the levels of autophagy-related proteins in human kidney tubular epithelial cells (Figure 4). In an *in vivo* study, KIM-1-TG NPs induced increased LC3 expression in kidney tissue (Figure 7B).

**Figure 8:**
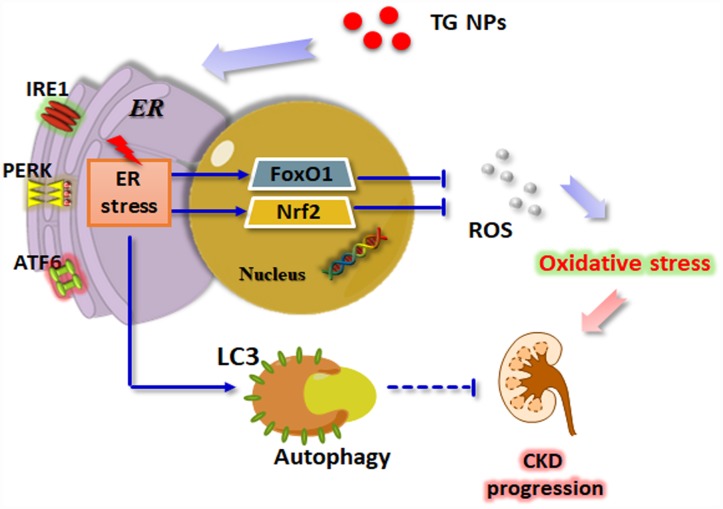
**Schematic model of how TG NPs ameliorate CKD progression.** TG NPs could induce the UPR pathway in kidney cells. Furthermore, TG NPs protected kidney cells against oxidative stress-induced cell death through the activation of Nrf2 and FoxO1. In addition, TG NPs induced autophagy that may inhibit CKD progression.

Taken together, these results show that the expression levels of ER stress- and autophagy-related genes were increased in the renal tissue of the CKD patients. We utilized nanotechnology to synthesize TG NPs that induced ER stress and the UPR pathway in HK-2 human kidney tubular epithelial cells (Figure 8). We demonstrated that the TG NPs protected HK-2 cells against oxidative stress-induced cell death through the activation of Nrf2 and FoxO1. Additionally, TG NP-induced autophagy may play a prosurvival role. In the model of adenine diet-induced kidney disease, the KIM-1-TG NPs ameliorated renal dysfunction and injury through the induction of ER stress and its downstream pathways. Molecules involved in the UPR pathway may offer new opportunities for pharmacological intervention against CKD in the future.

## MATERIALS AND METHODS

### Preparation of TG-encapsulated PLGA nanoparticles (TG-PLGA NPs)

Copolymers of poly(lactic-co-glycolic acid) (PLGA) (M.W. 45000 Da) with a 50/50 ratio of lactide to glycolide were purchased from Sigma-Aldrich Corp. (USA). All chemicals and reagents were analytical grade. A total of 50 mg of PLGA and 4 mg of TG (dissolved in acetone) were dissolved in 5 mL of acetone. Ethanol/H_2_O (50/50, %v/v) aqueous (the nonsolvent) was added dropwise into the PLGA solution and stirred at 400 rpm by a magnetic stirrer until turbidity appeared. Following stirring for 5 min, the suspension was transferred into 20 mL of deionized water in a glass beaker and stirred at 400 rpm for 20 min. The organic solvent was then removed. The collected suspension contained 74 ± 5.6 nm PLGA NP solution. The TG concentration of the TG-encapsulated PLGA NPs was measured using the following procedure: a bottle was weighed, and the weight was recorded before the TG-PLGA NPs or PLGA NPs were added. The TG-PLGA NPs or PLGA NPs were centrifuged at 13000 rpm for 10 min, and the supernatants were discarded. The resulting precipitates were washed with deionized water and dried under a vacuum for 24 h. The precipitate weight of the PLGA NPs was subtracted from that of the TG-PLGA NPs to obtain the weight of the entrapped TG. The encapsulation efficiency of the TG entrapment and the amount of TG in the PLGA NPs (% w/w) were then calculated.

### Preparation of antibody-conjugated TG-PLGA NPs (KIM-1-TG-PLGA NPs)

To immobilize antibodies on TG-PLGA NPs, 500 μL of TG-PLGA NPs was incubated with 500 μL of PBS (pH 7.4). Then, 25 μL of an anti-KIM-1 antibody (500 μg/mL) (R&D Systems, Minneapolis, MN) and 124 μL of 1-ethyl-3-(3-dimethylaminopropyl) carbodiimide hydrochloride (EDC) (100 mM) were added to the solution, which was then incubated at 4°C for 24 h before being centrifuged at 10000 rpm for 10 min. The supernatant was discarded, and the precipitate was washed with PBS.

### Characterization

Electron micrographs of TG-PLGA NPs were taken by placing a drop of the sample onto the copper mesh coated with an amorphous carbon film and drying the sample in a vacuum desiccator. The TG-PLGA NPs were stained with 1% aqueous sodium phosphotungstate solution before the transmission electron microscopy (TEM) images were acquired. The mean diameter and morphology of the TG-PLGA NPs were characterized using a transmission electron microscope (Hitachi Koki Co., Tokyo, Japan). The extinction characteristic of TG was measured using an ultraviolet-visible light (UV-vis) spectrophotometer (Agilent Technologies, Santa Clara, CA). The surface charge and hydrodynamic diameter of the TG-PLGA NPs were analyzed using zeta potential measurements (Malvern Instruments, Malvern, Worcestershire, UK).

### In vitro test of the release of TG from TG-PLGA NPs

To analyze the release of TG from TG-PLGA NPs, 100 μL of TG-PLGA NPs was separately incubated with 900 μL of phosphate-buffered saline (PBS) in Eppendorf tubes at 37°C for different time periods (1, 2, 4, 8, 12, 24, 36, 48, 72, 96, and 120 h). After these time periods, supernatants containing free TG released from the TG-PLGA NPs were collected by centrifugation at 13000 rpm for 10 min. A control set was created by using an equal amount of TG loaded into 1 mL of PBS buffer to obtain the TG concentration. The amount of released TG in each time period supernatant was determined using a UV-vis spectrophotometer. The ratio of the TG released from the TG-PLGA NPs to the TG in the control set was calculated based on the absorption intensity measured at the characteristic wavelength.

### Microarray analysis

Raw data from the microarray dataset GSE66494 was downloaded from the Gene Expression Omnibus (GEO) database and normalized with GeneSpring software as log_2_ values. Differential transcriptional activity between healthy individuals and CKD patients is presented as a boxplot, which was constructed using SPSS software.

### Cell culture

The human kidney proximal tubular epithelial cell line HK-2 was obtained from American Type Culture Collection (CRL2190) and cultured in Keratinocyte Serum-Free (KCSF) medium with recombinant epidermal growth factor (5 ng/ml) and bovine pituitary extract (40 μg/ml) (Gibco BRL, NY) at 37°C and 5% CO_2_.

### Cell viability assay

Cell viability was analyzed using a sulforhodamine B (SRB) assay. Cells were plated in 96-well plates. After incubation, the cells were fixed with a trichloroacetic acid solution for 1 h, and SRB (Sigma-Aldrich Corp.) was added to each well for 1 h. The plates were washed and Tris buffer (20 mM) was added. Then, the absorbance of the solution was read at 562 nm on an ELISA reader (Molecular Devices, Sunnyvale, CA). The mean absorbance of the untreated cells was used as the reference value for calculating 100% cell viability.

### Western blot analysis

Total protein was prepared from cell lysates by harvesting cells in protein extraction buffer at 4°C for 1 h. The proteins isolated from the cells were loaded at 30 μg/lane along with HR Pre-Stained Protein Marker 10–170 kDa (BIOTOOLS, Taiwan) on a TOOLS HR Gradient Gel (BIOTOOLS, Taiwan), subjected to electrophoresis, blotted, probed using antibodies and detected using a chemiluminescence (ECL) detection system (Thermo Fisher Scientific, Waltham, MA). Anti-Beclin 1, anti-p-eIF2α, anti-eIF2α, anti-GAPDH, anti-IRE1α and anti-LC3 antibodies were obtained from Cell Signaling Technology (Ipswich, MA, USA); anti-ATF6, anti-FoxO1, anti-NRF2 and anti-ATG5 antibodies were obtained from Proteintech Group (Chicago, IL, USA); and an anti-p84 antibody was obtained from Bioss Antibodies Inc. (Woburn, MA, USA). Nuclear and cytoplasmic fractions were collected using the Nuclear/Cytosol Fractionation Kit from BioVision Inc. (Milpitas, CA, USA), according to the manufacturer’s instructions. The densities of the bands were quantified with a computer densitometer (AlphaImager™ 2200 System Alpha Innotech Corporation, San Leandro, CA, USA).

### Immunofluorescence microscopy

Cells were cultured on coverslips. After TG NP treatment, the cells were fixed in paraformaldehyde (4%) and blocked with BSA (1%) for 30 min. This step was followed by incubation with an anto-LC3 antibody (MBL, Japan) for 1 h. After washing, the cells were labeled with DyLight™ 488-conjugated AffiniPure goat anti-rabbit IgG (Jackson ImmunoResearch Laboratories, PA, USA) for 1 h and stain with 4',6-diamidino-2-phenylindole (DAPI) (Invitrogen, USA). Finally, the cells were washed in PBS and examined with a fluorescence microscope or confocal microscope (Leica TCS SP5, Mannheim, Germany).

### Transmission electron microscopy (TEM)

Cells were harvested and the fixed for 1 h in a solution containing 2.5% glutaraldehyde and 2% paraformaldehyde in 0.1 M cacodylate buffer. After fixation, the samples were postfixed with 1% OsO_4_ for 30 min. Ultrathin sections were examined using a transmission electron microscope (Hitachi HT-7700, Tokyo, Japan).

### RNA Interference (RNAi)

We utilized the *Trans*IT-X2® Dynamic Delivery System (Mirus, WI, USA) to transfect cells according to the manufacturer’s protocol. Briefly, Opti-MEM I reduced-serum medium, TransIT-X2 and an siRNA solution were pipetted gently to mix completely. The mixed solution was incubated at room temperature for 30 min to allow sufficient time for complexes to form. Then, TransIT-X2:siRNA complexes were added to different areas of wells containing cells for 24-72 h. FoxO1 (ID: SASI_Hs01_00076737) and NRF2 (ID: SASI_Hs01_00182393) siRNAs were obtained from Sigma-Aldrich (St. Louis, MO, USA).

### Adenine diet-induced CKD

Eight-week-old male C57BL/6 mice were acquired from the BioLASCO Experimental Animal Center (Taiwan). The animals were housed five per cage with 50% ± 10% relative humidity at 24 ± 2°C and subjected to a 12-h light/dark cycle. The animals were acclimatized for 1 week prior to the start of experiments. The mice were fed a Purina chow diet with water ad libitum. All experiments with mice were performed according to the guidelines of our institute (the Guide for the Care and Use of Laboratory Animals, Taipei Medical University). The animal use protocol listed below was reviewed and approved by the Institutional Animal Care and Use Committee of Taipei Medical University, Taiwan. The mice were randomly divided into the following six groups (five animals/group): chow-fed mice (Normal), adenine-fed and intraperitoneally saline-injected mice (CKD), adenine-fed and intraperitoneally low-concentration (0.1 mg/kg) TG NP-injected mice (CKD+TL), adenine-fed and high-concentration (0.2 mg/kg) TG NP-injected mice (CKD+TH), adenine-fed and low-concentration (0.1 mg/kg) KIM-1-TG NP-injected mice (CKD+KTL) and adenine-fed and high-concentration (0.2 mg/kg) KIM-1-TG NP-injected mice (CKD+KTH). To induce CKD, the mice were fed a 0.2% adenine-containing diet for a period of 5 weeks. In the drug treatment groups, the mice were fed an adenine-containing diet for 1 week and then were intraperitoneally injected with the NPs two times per week for 4 weeks while being fed the adenine-containing diet. The mice were sacrificed via CO_2_ exposure. After euthanasia, the kidney tissue of the mice was formalin fixed and paraffin embedded for immunohistochemistry (IHC).

### Biochemistry tests

Whole blood samples were collected by intracardiac puncture and centrifuged at 2000 × g for 20 min to separate the serum. The biochemistry evaluation included the serum urea nitrogen and creatinine levels.

### Histopathological and immunohistochemical staining analyses

Paraffin-embedded kidney tissue sections were dried, deparaffinized, and rehydrated. Following microwave pretreatment in citrate buffer (pH 6.0), the slides were immersed in hydrogen peroxide (3%) for 20 min to block the activity of endogenous peroxidases. Histopathological observations were performed with hematoxylin and eosin (H&E)-stained slides. Tissue sections were incubated overnight at 4°C with anti-LC3 (MBL, Japan), anti-FoxO1 (Proteintech Group, USA) or anti-NRF2 (Proteintech Group, USA) antibodies. The sections were then incubated with a secondary antibody for 1 h at room temperature, and the slides were developed using a STARR TREK Universal HRP detection kit (Biocare Medical, Concord, CA). Finally, the slides were stained using hematoxylin.

### Statistical analysis

We assessed the differences in continuous variables (presented as the mean ± standard deviation [SD]) between groups using a two-sample t-test or one-way analysis of variance combined with a post hoc Bonferroni test. All statistical analysis were performed at a two-sided significance level of 0.05.

## Supplementary Material

Supplementary Figures
