# Supplementary material for: Promising therapeutic effect of thapsigargin nanoparticles on chronic kidney disease through the activation of Nrf2 and FoxO1
Source: Aging (Albany NY). 2019 Nov 12;11(21):9875–92. doi: 10.18632/aging.102437 (PMC6874456; doi:10.18632/aging.102437)
Supplement: Supplementary Figures [file aging-11-102437-s001.pdf]

SUPPLEMENTARY FIGURES

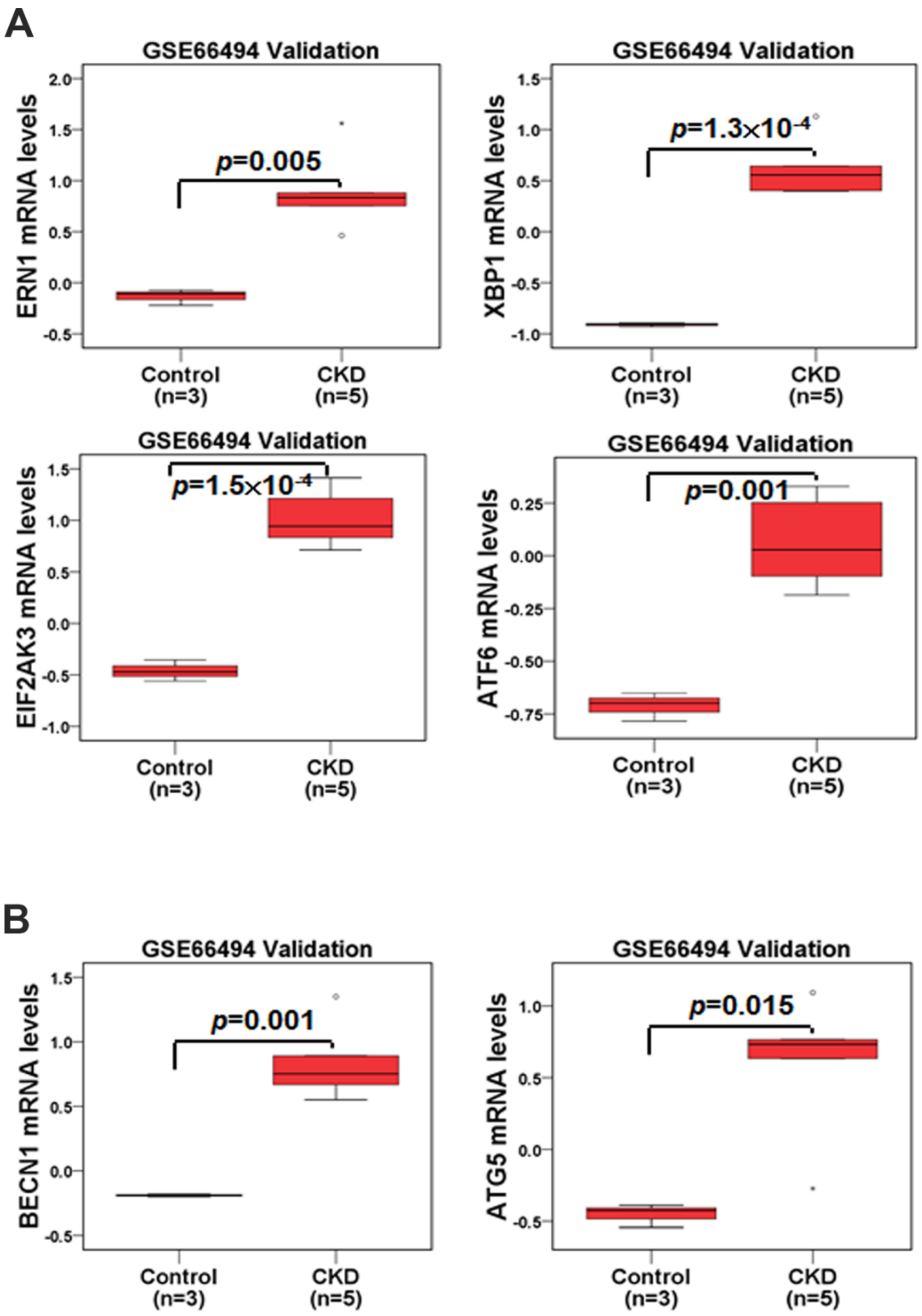

Supplementary Figure 1. The mRNA levels of ER stress- and autophagy-related molecules were analyzed in the renal tissue of healthy individuals and CKD patients. The mRNA levels of *ERN1*, *XBP1*, *EIF2AK3*, *ATF6* (**A**), *BECN1* and *ATG5* (**B**) were evaluated. Statistical differences were analyzed using a two-sample t-test.

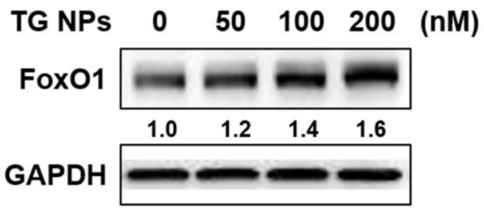

Supplementary Figure 2. The protein levels of FoxO1 in HK-2 cells treated with TG NPs are shown. Cells were treated with different concentrations of the TG NPs for 24 h.
